# HOW COVID-19 NEWS AFFECTS OLDER ADULTS' MENTAL HEALTH

**DOI:** 10.1093/geroni/igac059.1953

**Published:** 2022-12-20

**Authors:** Grace Li, Zoe Ziyi Ng, W Quin Yow

**Affiliations:** Palo Alto High School, Palo Alto, California, United States; Raffles Institution, Singapore, Singapore; Singapore University of Technology & Design, Singapore, Singapore

## Abstract

Media affects the trajectory of many individuals’ mental health. With the rise of COVID-19 cases, the media not only provides informative updates on the pandemic, it also fuels the hysteria and paranoia of citizens across the world. Studies show that older adults (OA) who consume media frequently were at a higher risk for declining mental health (Negarestani et al., 2021) and that individuals experience negative bias more than positive bias (Vaish et al., 2013). However, no studies have directly investigated how much positive and negative COVID-19 news affect OA’s mental health and emotions. Sixty-nine OA (aged 55-95) answered questions about their weekly media consumption, how closely they followed COVID-19 news, and the General Health Questionnaire. They were then randomly assigned to read either positive or negative COVID-19 news (n=35 and 34 respectively), and asked if the news made them feel happy/fearful and if they wanted to read more about/ignore the news. Analysis revealed that the more OA consumed media and closely followed COVID-19 news, the more they felt unhappy and depressed, rs>.25, ps<.05. There was a significant condition effect, where OA who read positive news reported more positive emotions than those who read negative news, t(67)=5.21, p<.001. Chance analyses revealed that positive news evoked positive emotions significantly above chance, t(34)=8.99, p<.001, but negative news evoked negative emotions only marginally above chance, t(33)=1.92, p=.064. These findings suggest that media consumption of COVID-19 news does negatively impact OA’s mental wellbeing, and OA appear to have a strong positivity bias for COVID-19 news.

